# Henipavirus Infections: Lessons from Animal Models

**DOI:** 10.3390/pathogens2020264

**Published:** 2013-04-08

**Authors:** Kévin P. Dhondt, Branka Horvat

**Affiliations:** 1 International Center for Infectiology Research, INSERM U1111, CNRS UMR5308, Ecole Normale Supérieure de Lyon, University of Lyon 1, 21 Avenue T. Garnier, Lyon 69007, France; E-Mail: kevin.dhondt@inserm.fr; 2 International Center for Infectiology Research, INSERM U1111, CNRS UMR5308, Ecole Normale Supérieure de Lyon, University of Lyon 1, 21 Avenue T. Garnier, Lyon 69007, France; E-Mail: branka.horvat@inserm.fr

**Keywords:** emergent infections, zoonosis, pathogenesis, animal models

## Abstract

The *Henipavirus* genus contains two highly lethal viruses, the Hendra and Nipah viruses and one, recently discovered, apparently nonpathogenic member; Cedar virus. These three, negative-sense single-stranded RNA viruses, are hosted by fruit bats and use EphrinB2 receptors for entry into cells. The Hendra and Nipah viruses are zoonotic pathogens that emerged in the middle of 90s and have caused severe, and often fatal, neurologic and/or respiratory diseases in both humans and different animals; including spillover into equine and porcine species. Development of relevant models is critical for a better understanding of viral pathogenesis, generating new diagnostic tools, and assessing anti-viral therapeutics and vaccines. This review summarizes available data on several animal models where natural and/or experimental infection has been demonstrated; including pteroid bats, horses, pigs, cats, hamsters, guinea pigs, ferrets, and nonhuman primates. It recapitulates the principal features of viral pathogenesis in these animals and current knowledge on anti-viral immune responses. Lastly it describes the recently characterized murine animal model, which provides the possibility to use numerous and powerful tools available for mice to further decipher henipaviruses immunopathogenesis, prophylaxis, and treatment. The utility of different models to analyze important aspects of henipaviruses-induced disease in humans, potential routes of transmission, and therapeutic approaches are equally discussed.

## 1. Introduction

The Hendra and Nipah viruses (HeV and NiV) are recently emerged zoonotic viruses responsible for outbreaks of respiratory and neurological disease in livestock and humans. Both are negative-sense single stranded RNA viruses and belong to the *Henipavirus* genus within the *Paramyxoviridae* family [1]. Very recently, a third member, the Cedar virus, joined the *Henipavirus* genus. While HeV and NiV infection are known to cause very high mortality rates, ranging between 40 and 100% in both humans and animals [2,3], the Cedar virus seems to be nonpathogenic. Henipaviruses genomes are among the largest of the *Paramyxoviridae* family, exceeding 18kb, with sequence homology between HeV and NiV ranging from 78 to 92%, and between HeV and Cedar virus from 28% to 58% [4]. Henipaviruses virions consist of six structural proteins. The genomic RNA is enclosed by nucleocapsid proteins, which together with phosphoprotein and RNA polymerase large protein forms the transcriptase complex. The viral envelope is formed by matrix proteins associated to a lipid bi-layer and two surface glycoproteins, glycoprotein and fusion protein, which allow, respectively, viral attachment and entry into the host cell. As in other paramyxoviruses, P gene encodes for non-structural proteins: C protein from the alternative open reading frame and W and V proteins, following RNA transcriptional editing, although this editing ability of viral polymerase seems to be absent in the Cedar virus [4]. 

HeV was identified as the causative agent of an outbreak of acute respiratory disease in humans and thoroughbred horses in Brisbane, Australia in 1994 [5]. Since its first appearance, HeV has re-emerged several times and presents a serious threat to horse livestock in Australia, with sporadic and lethal transmission to humans. NiV was first recognized following a 1998-99 outbreak of severe febrile encephalitis in Malaysia and Singapore [6,7]. Subsequent outbreaks of NiV occurred in India and almost annually in Bangladesh [8,9,10], where the sequence of Bangladesh isolate of NiV was shown to differ slightly from the Malaysia isolate [11]. Comparison between Malaysia and Bangladesh isolates revealed a nucleotide variation range of 6.32%–9.15% and an amino acid variation range of 1.42%–9.87% [12]. Survivors of acute encephalitis may experience relapse encephalitis years after initial infection and people that get infected asymptomatically may develop a late onset disease [13]. Cases of inter-human transmission of NiV-Bangladesh further extends its potential to cause deadly outbreaks [14,15]. Furthermore, the distribution area of henipaviruses and henipa-like viruses ranges outside Australia and Asia. Serological evidence found in African fruit bats [16] suggests the circulation of henipa-like viruses across this continent and new viruses will probably join this genus in the future. The ability to cause serious human and animal diseases which can be transmitted from one individual to another, and the lack of efficient treatment and preventive measures, led to the classification of HeV and NiV as biosecurity level 4 (BSL-4) pathogens. 

The cellular and molecular basis of high pathogenicity of HeV and NiV is still far from being understood. The development of adequate animal models is required for better comprehension of *Henipavirus* infection and their transmission as well as for the generation of efficient prevention and treatment. As these viruses are zoonotic, there are a number of different animal species susceptible to infection. Some of them are natural hosts and their investigation could provide useful information about virus replication, shedding, and transmission. Laboratory animal models were developed to evaluate vaccines and anti-viral treatments. Finally, the relevance of different models for understanding the infection in humans varies among models. This review discusses the major characteristics of animal models used to study HeV and NiV infection and summarizes the principal features of viral pathogenesis in the most commonly used models (Table 1).

## 2. Fruit Bats

There is increasing evidence that bats play a major role in the emergence and transmission of new and deadly zoonotic viruses [62,63]. Fruit bats (Pteropus species) are putative natural reservoir hosts for a number of viruses, including NiV, HeV, and the Ebola virus. Although these viruses often cause asymptomatic infections in their natural host, once they cross the species barrier they can become extremely virulent with high mortality rates. Serological studies have shown that the seroprevalence of antibodies to henipaviruses in pteropid bats varies from 10% to 50% in numerous countries, including Australia [64,65], Malaysia [66], India [67], Cambodia [68,69], Indonesia [70], and Thailand [71]. Subsequent analysis showed serological evidence for henipaviruses in China [72], Madagascar, Papua New Guinea, and Ghana [73], thus, expanding significantly the known distribution of these viruses. HeV was initially isolated from uterine fluid and fetal tissue of a *Pteropus policephalus* and *P. alecto* [65]. NiV was isolated from urine of *P. hypomelanus* in Malaysia [74], and *P. lylei* in Cambodia [69]. Analysis of captive *P. vampyrus* showed high seroprevalence and horizontal transmission of the Nipah virus [75]. Finally, a new member of *Henipavirus* genus, the Cedar virus, was recently isolated from urine samples from Australian flying foxes [4].

Experimental infection of fruit bats with HeV and NiV virus was followed by seroconversion in the majority of animals but showed absence of visible clinical disease, despite the inoculation of rather high viral doses. HeV infection of grey-headed fruit bats (*P. poliocephalus*) resulted in vascular lesions, positive immunostaining, and virus isolation [18]. Virus was also recovered from the fetus, confirming that pathogenesis in pregnant animals may result in viral crossing of the placenta, supporting thus the possibility of vertical transmission. However, transmission of HeV from bats to horses could not be experimentally demonstrated [18]. Experimental subcutaneous NiV infection in grey-headed fruit bats resulted in generation of neutralizing antibodies and histological changes in different tissues. The isolation of the virus was possible from the urine, kidney, and uterus [17]. Oronasal *Henipavirus* infection of bats gave only occasional seroconversion. Virus isolation was usually not possible, although HeV was isolated from one *P. alecto* female in that study [19]. Occasional low-level excretion of henipaviruses in the urine of bats may be sufficient to sustain virus circulation in bat colonies. Nevertheless, the rare and sporadic spillovers seem to require the coincidence of a range of individual and environmental factors. Taken together, these studies demonstrate that fruit bats can carry henipaviruses, without the manifestations of clinical disease or any gross pathology, in contrast to most of other mammalian species. However, the mechanism responsible for the efficient control of viral replication in bats remains to be elucidated. Understanding how bats coexist with viruses has important implications in predicting spillover events from bats to other susceptible species.

**Table 1:** Principal characteristics and current utilization of animal models of NiV (white fields) and HeV (grey fields) infection. +++ advised model, ++ highly suitable model, + suitable model, +/- better models existing, - model not recommended.

Species	Clinical signs			Gross lesions	Histology	Virus found in	Utilisation of the model					References
	Respiratory		Neurological				Patho-logy	Immu-nology	Drugs assess-ment	Vaccine assess-ment	Epide-miology	
Fruit bat	-		-	petechial hemorrhages on urine bladder wall	granulomatous hepatitis, inflammation of bladder epithelium, vasculitis, testicular degeneration	kidney, urine, uterus	-	+	-	-	+++	[17]
	-		-	-	necrosis and hemorrhage of the adrenal gland	urine, rectal swab						[18,19]
Pig	severe non-productive cough		shivering, seizures	lymph nodes hyperplasia, congestion, edema	hyperplasia of BALT, meningitis, encephalitis	lymphoid organs, CNS, lung	+	+	++ (vet. use)	++ (vet. use)	+++	[20,21,22,23,24]
	cough, respiratory distress		incoordination (transient)	congestion, hemorrhages	syncytial cells in nasal turbinates and bronchiolar epithelium	tonsils, BALF, lung, nasal turbinates, lymph nodes, swabs						[25]
Horse	-		-	-	meningitis	-	+	-	++ (vet. use)	++ (vet. use)	+++	[26]
	increased respiratory rate, frothy nasal discharge		ataxia, head pressing, myoclonic twitches	congestion, edema, hemorrhages in lung	interstitial pneumonia, vasculitis, syncytia in endothelium	brain, lung, lymphoid organs, kidney, bronchial and oral swabs, urine						[5,18,27,28]
Cat	tachypnea, dyspnea		-	congestion ,edema, hemorrhages, enlarged lymph nodes	acute bronchiolitis, necrotizing alveolitis, vasculitis, endothelial syncytia	lung, spleen, kidney, lymph nodes	+/-	-	+	++	-	[21,29,30,31]
	dyspnea, open mouth breathing		-	hydrothorax, congestion, edema, hemorrhages	inflammation in lung, alveolar wall necrosis, vasculitis, endothelial syncytia	lung, spleen, lymph nodes						[18,32,33,34]
Ferret	dyspnea, cough, serous nasal discharge		depression, tremors, myoclonus, hind limb paresis	edema, hemorrhages, enlarged lymph nodes	necrotizing alveolitis, glomerular necrosis, vasculitis, endothelial syncytia, meningitis	brain, lung, lymphoid organs, adrenal, kidney, testes, uterus, liver, pharyngeal and rectal swabs	+	-	++	++	-	[35,36,37,38]
	-		depression, tremors			lung, lymphoid organs, adrenal, meninges, kidney, liver, testes, oral and rectal swabs, blood, urine						[39]
Squirrel Monkey	dyspnea, tachypnea		ataxia, coma, seizures	not reported	pulmonar inflammation, mild vasculitis	lung, brain, kidney, spleen	+	+	+/-	+/-	-	[40]
African Green Monkey	severe dyspnea, open-mouth breathing, serosanguineous nasal discharge		muscle twitches, behavioral changes, loss of balance	pleural effusion, congestion and hemorrhage in lungs, hemorrhages on mucosal surface of urinary bladder, edema and hemorrhages of the meninges	endothelial syncytial cells, vasculitis, meningitis, inflammation, fibrinoid necrosis	liver, spleen, kidney, adrenal gland, lung, lymph nodes, pancreas, sex organs, urine, nasal swabs	+++	+	+++	+++	-	[41,42]
	nasal discharge, labored breathing		muscle twitches, seizures	pulmonary consolidation, congestion of lungs, enlarged lymph nodes, congested liver, inflammation of gastrointestinal tract, congestion in the brain	alveolar hemorrhages, pulmonary edema and inflammation, alveolitis, fibrinoid necrosis, vasculitis, meningitis	lung, lymph nodes, heart, liver, spleen, kidney, adrenal gland, brain, urinary bladder, sex organs						[43,44]
IFNAR KO mouse		-	behavioral troubles, ataxia, pain, paralysis	brain and lung congestion, edema of bladder wall, hemorrhages, necrosis in liver and kidney	vasculitis, inflammation, meningitis, encephalitis, necrotizing alveolitis	brain, lung, spleen, liver	+	+++	+++	+	-	[45]
		-		brain and lung congestion, hemorrhages	vasculitis, meningitis, encephalitis, gliosis, necrotizing alveolitis	brain, lung, spleen, liver						[45]
Aged mouse		-	-	-	-	-	+/-	+	+	-	-	-
		-	ataxia, muscle tremors	-	encephalitis, meningitis	brain, transiently in lung						[46]
Hamster		labored breathing, serosanguineous nasal exudates	imbalance, muscle twitching, tremor, limb paralysis	edema, hemorrhages, congestion	vasculitis, meningitis, encephalitis, endothelial syncytia	lung, nasal epithelium, CNS, heart, liver, spleen, kidney, bladder, urine	+++	+/-	++	++	+	[47,48,49,50,51,52,53,54,55,56,57,58]
Hamster		acute respiratory distress, serosanguineous nasal discharge	imbalance, ataxia, muscle twitching, limb paralysis	inflammation and edema of the lungs	inflammation, alveolitis, necrotizing vasculitis, meningitis, encephalitis, hemorhage in the brain, neuronal necrosis, gliosis, endothelial syncytial cells	spleen, kidney, heart, lung, brain, liver						[48,56,58,59]
Guinea pig		-	mild behavioral changes, ataxia	mesenteric edema	vasculitis, lymphoid depletion, endothelial syncytia with fibrinoid necrosis, edema, hemorrhage and ulceration of the urinary bladder, meningitis	heart, kidney, lymph nodes, uterus	-/+	-	-	-	-	[17]
		-	head tilt	congestion, cyanosis, edema	pulmonar fibrinoid necrosis, vasculitis, endothelial syncytia, encephalitis	brain, kidney, urine, uterus, placenta						[34,60,61]

## 3. Farm and Domestic Animals

Despite the poor ability of most *Paramyxoviridae* to cross species-barriers, henipaviruses are able to infect a broad range of mammalian species. The susceptibility of several mammalian species to natural infection with NiV and HeV is mainly responsible for their emergence in Southeast Asia and re-emergence each year in Australia and Bangladesh. This observation may be explained by the high conservation of the viral entry receptors, Ephrin (EFN) B2 and B3, amongst mammalian species. Indeed, protein sequence analysis revealed that identity between human and horse, pig, cat, dog, *Pteropus* bat, and mouse EFN B2 and B3 ranges from 95% to 98% [76]. Contrarily to NiV and HeV that can use both EFN B2 and B3 receptors [77], the Cedar virus was shown to use EFN B2 receptor only [4].

Farm and domestic animals often play the role of intermediate hosts between the wild reservoir and the human population. Rapidly, after viral emergence, pigs and horses were found to be amplifying secondary hosts, respectively for Malaysia NiV strain and HeV. Yet, looking for secondary reservoirs a broad range of animal species were serologically tested. Birds [9,78], peri-domestic and domestic rodents [9,66,78,79], wild boars, and insectivorous bats [66] tested negative. Both cats and dogs were found to be susceptible to NiV infection even if there were only two field cases for dogs and one field case for cats [26]. Studies showed that a very close contact with infected pigs is necessary for infection of these species. Indeed, feral cats [80] and dogs [81] from non-infected pig farms, but in contact with potentially infected fruit bats, were seronegative. In Bangladesh, human cases were associated with contact with sick cows [9] and goats [15], but no clinical or serological studies were able to confirm this transmission. In Australia, the only natural HeV infection in a species other than horses and bats was reported in 2011 and concerned a healthy seropositive dog from an infected property [82].

### 3.1. Pigs

In a natural context, NiV infection in pigs most likely occurs from oronasal contact with infected urine, faeces, or saliva from bats. The spreading of NiV from pig to pig is very high with a morbidity rate close to 100%, whereas the lethality rate varies between 1% to 40% depending on the age of the animal. Infection is usually asymptomatic, but some pigs showed respiratory signs characterized by a severe nonproductive cough (“barking pig syndrome”) and sudden death [20]. The study of NiV infection in pigs in an experimental context was rapidly carried out after the discovery of the virus. Oronasal and ocular routes mimic, quite well, natural infection and most infected animals remain asymptomatic [21,22,23] even if viral shedding is observed in nasal and pharyngeal swabs from three to seven days post-infection [23]. Sick animals present fever, depression, cough, shivering, and rarely, neurological signs including abnormal posture [24] and seizures [23]. Subcutaneous route was also tested with an increase in severity and frequency of respiratory and neurological signs [21]. Gross pathology findings include hyperplasia of submandibular, mesenteric, and bronchial lymph nodes, congestion and oedema of meninges [24] and lung consolidation. Histology reveals hyperplasia of BALT, non-suppurative meningitis, and encephalitis [21]. Immunohistology shows evidence of invasion of the central nervous system through olfactory nerves [23]. Infected pigs are able to develop neutralizing antibodies to high titers within two weeks, post-infection. In Africa, cross-reactive but not cross-neutralizing antibodies to HeV or NiV were found in pigs, confirming the presence of circulating henipa-like viruses in this geographic area [83].

Studies based on NiV infection of pigs provided new insights of the role of peripheral blood mononuclear cells. In contrast to what has been seen in human lymphocytes [84], certain populations of swine lymphocytes seem to be permissive to NiV infection [85]. Piglets that succumbed to NiV infection had a significant drop in the CD4+CD8− T cell frequency compared to those that survived. As these T helper lymphocytes aid the development of humoral responses, this observation supports the hypothesis that a rapid production of virus-specific antibodies is necessary for a fully protective anti-NiV response [85]. Moreover, experimental infection of piglets with NiV induced a lymphoid depletion in lymph nodes that was responsible for subsequent bacterial infection of immunodepressed animals [24]. Lymphotropism remains so far a unique attribute of NiV infection in swine and may account for some features of pigs as a spillover host. 

The only attempt to infect pigs with HeV reports that nasal and oronasal inoculation usually induce respiratory symptoms both in farm pigs and minipigs. Some neurological signs (incoordination) were transiently observed on Gottingen minipigs [25]. At necropsy, a congestion of lungs and petechial hemorrhages on multiple organs were observed. Syncytial cells were frequently noticed in the respiratory epithelium of nasal turbinates and the bronchiolar epithelium. Viral RNA was detected in decreasing order for nasal, oral, rectal, and ocular swabs. Virus was successfully isolated from tonsils, bronchial-alveolar lavage fluid (BALF), lung and olfactory bulbs, nasal turbinates, submandibular and bronchial lymph nodes and swabs. Pigs developed neutralizing antibodies from day five to seven after infection.

### 3.2. Horses

Horses were used as a first animal model of henipaviruses infection, to confirm that HeV (called equine morbillivirus in that period) is the etiological agent of the new infectious disease [5]. The clinical course of HeV, in both naturally and experimentally infected horses, is relatively short, with death occurring within 48 h after the onset of clinical signs. Experimental studies reveal an incubation period ranging from five to 10 days following infection [5,18,27]. Whatever the route of infection (intranasal [5,18,27], intravenous [5], or subcutaneous [18]), affected horses usually present a febrile syndrome (>40 °C), depression, increased respiratory and heart rates, and as the disease progresses, frothy nasal discharge and neurological disabilities such as ataxia, head pressing [28], and myoclonic twitches [18]. Some horses seroconverted but still remained asymptomatic following subcutaneous [18] or natural [5] infection. Gross pathology is preferentially found in the lung area where congestion, edema [5], dilatation of the lymphatics [18], and subpleural hemorrhages [27] were shown. Histological examination usually confirms an interstitial pneumonia and a severe systemic vasculitis [5,18,27]. The presence of syncytial giant cells is detectable in lungs, kidney, lymph nodes [27], and mostly concerns endothelial cells of the blood vessels [5]. Viral isolation has been successfully performed from bronchial and oral swabs, urine as well as from brain, blood, spleen, lung, kidney, and lymph nodes [18]. Although the recovery of viral particles was not possible in rectal swabs and feces, viral RNA was found by RT-qPCR analysis [27]. Despite viral shedding in many fluids, transmission of HeV between horses remains an unlikely event [18] and seems to require very close contact in natural conditions [86,87].

Concerning NiV, there is only one natural confirmed equine case. Histopathology on this case revealed non suppurative meningitis associated with several areas of cell depletion in the parenchyma of the brain [26].

### 3.3. Cats

The cat (*Felis sylvestris catus*) is a receptive animal model for both HeV and NiV infection. Rapidly after the discovery of HeV in 1994, the cat was tested for its susceptibility to infection [32]. Cats infected either by subcutaneous, intranasal, or oral routes become clinically ill within four to eight days [33,34]. The illness is restricted to a pulmonary syndrome with severe dyspnea and open mouth breathing [18,33]. The gross pathology is mainly focused in the lungs with hydrothorax and pulmonary edema associated with congestion and intrapulmonary hemorrhages [34]. The bronchial and mesenteric lymph nodes are enlarged, pale, and sometimes present petechial hemorrhages. The spleen is also enlarged. Histopathology of the lung is consistent with gross findings and confirmed the edema and hemorrhages. These observations are completed with a high number of alveolar macrophages, alveolar wall necrosis, and vascular lesions including thrombosis, fibrinoid necrosis, and endothelial syncytia [34]. Transmission of the virus is possible from cat to cat [33], and from cat to horse [18] but remains a relatively rare event. 

The use of cats to study NiV was also assessed using either intranasal [21] or subcutaneous routes [29]. The clinical signs are relatively similar to those observed following HeV infection and include febrile syndrome, mental depression, tachypnea, and dyspnea [21,29]. Necropsy and histology indicate similar results to those found for HeV. Infectivity of cats via oronasal route was not assessed for NiV, but the vertical transmission from the female to the fetus was demonstrated. Placenta, uterine fluids, and fetal tissues were positive for NiV replication. The placenta and placental fluids contained a significant titer of virus [30] thus indicating a potential role of pregnancy in the shedding of the virus by fruit bats. Although the cat is a very susceptible model for henipaviruses infection, its utilization to study human pathology is still controversial as it does not present the neurological signs observed in humans.

The cat model was used to assess the potential of a vaccine that uses the recombinant soluble form of either HeV or NiV G protein, to protect against NiV infection [29]. Cats generated a good IgG and IgA antibody response towards NiV, especially with sHeV G subunit vaccine [31], and all vaccinated animals survived a NiV challenge.

### 3.4. Ferrets

Ferrets (*Mustela putorius*) are a suitable model for many respiratory diseases, including *Henipavirus* infection. Oronasally NiV infected ferrets develop clinical signs within six days post-infection including fever, severe depression, tremors, myoclonus [35], and hind limb paresis [36,37] but also respiratory signs such as dyspnea, cough, and serous nasal discharge [38]. Clinical signs of HeV infected animals are restricted to fever, depression, and generalized tremors from day six, post-infection, whatever the dose inoculated (from 50 to 50000 TCID_50_) [39]. Gross lesions are similar for both henipaviruses and show subcutaneous edema, petechial hemorrhages throughout the skin, the pulmonary parenchyma and the abdomen but also enlarged and hemorrhagic submandibular, retropharyngeal, bronchial, duodenal, and mesenteric lymph nodes [36,37,38,39]. Histopathology performed on infected tissues reveals acute focal necrotizing alveolitis and glomerular necrosis, systemic vasculitis with prominent endothelial syncytia, and focal necrosis of the spleen [38]. Non-suppurative meningitis was noticed in animals that presented neurological signs [38]. HeV antigens were identified in meningeal endothelial cells [39] and NiV antigens were found in the brain parenchyma [38]. Viral shedding was demonstrated by quantitative PCR from all fluids [37] especially in blood, pharyngeal and rectal swabs [38] and urine [39]. Viral isolates were successfully recovered from feces and urine in one study, especially between day seven and nine post-infection [35]. Comparative analysis between Malaysia and Bangladesh strains of NiV shows similar clinical signs. The Malaysia strain-infected ferrets present however a more pronounced hemorrhagic state. Concerning transmission of the virus, analysis of oral shedding reveals the Bangladesh strain to be more excreted than the Malaysia strain [35]. 

Ferrets were used to assess the efficiency of chloroquine as potential treatment of NiV infection, where no significant effect was observed [37]. They were then used in the successful evaluation of passive [38] or active [39] immunotherapy respectively based on human monoclonal antibodies or recombinant HeV G glycoprotein-based subunit vaccine. Neutralizing antibodies from ferrets infected by the Cedar virus were detected as early as 10 days post-infection and at high titer 21 days post-infection although the ferrets remained clinically healthy [4].

## 4. Non-Human Primate Models

**Figure 1:**
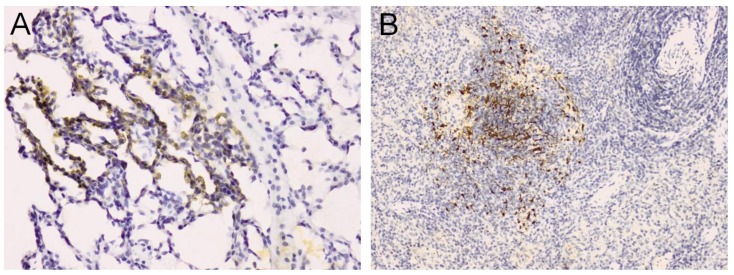
Immunohistochemistry analysis from NiV-infected Squirrel Monkeys. (**a**) Lung. Inflammation associated with positive immunostaining (brown) in the alveolar wall; (**b**) Spleen. Positive immunostaining (brown) located in the white pulp. Original magnification 20×.

The development of primate models is necessary for preclinical tests of preventive and therapeutic anti-viral approaches. The first nonhuman primate model described for NiV infection was the Squirrel monkey (*Saimiri sciureus*) [40]. Clinical illness with an acute neurological and respiratory syndrome associated with high mortality was observed in intravenously infected monkeys. Viral RNA and antigens were detected in different tissues (Figure 1). High antibody-neutralization titers were found only in monkeys that survived infection. Similarly to what has been shown in hamsters, free virus was not found in plasma of Squirrel monkeys, but rather virus was associated with the cell compartment, in accordance with the hypothesis that NiV could use circulating cells as carriers for efficient transmission within a host [84]. As observed in human infection of the Malaysian outbreak [88], only half of the challenged animals exhibited clinical signs, making this model potentially interesting to understand NiV pathogenesis in humans, although less convenient, compared to the other described animal models for the assessment of anti-viral approaches. 

In a second primate model, African green monkeys (AGM) were evaluated for both NiV and HeV infection [41,43]. Virus challenge by intratracheal inoculation, of either NiV or HeV, resulted in a rapid spread of the virus and infection of multiple organs, leading to high mortality. Severe respiratory pathology, neurological disease, endothelial syncytia, and generalized vasculitis were observed with both viruses. Immunohistochemical analysis showed the presence of viral antigens in different organs including lungs, the brainstem, and spleen.

The AGM model was shown to be a useful tool to evaluate antiviral therapy and prophylaxis in primates. The efficiency of ribavirin as a prophylactic and postexposure treatment was evaluated in HeV infection, demonstrating that it could delay disease onset without having significant benefit for disease progression and outcome [43]. Passive and active immunization against HeV and NiV, respectively, were proven to be successful in this model with the use of a recombinant human monoclonal antibody possessing virus-neutralizing properties [44] and a G glycoprotein subunit vaccine [42].

## 5. Rodent Models

### 5.1. Golden Syrian Hamster

The Golden Syrian Hamster (*Mesocricetus auratus*) is a commonly used model to study henipaviruses pathogenesis as the clinical signs and pathological lesions observed following intranasal or intraperitoneal inoculation highly resemble those observed in humans. When infected with NiV, the golden hamster shows a progressive behavioral deterioration, lethargy, imbalance, muscle twitching, tremors, and limb paralysis [47]. At high doses (10^5^ TCID_50_), and particularly when inoculated intranasally, the clinical signs are mainly restricted to an acute respiratory syndrome with labored breathing and serosanguineous nasal exudates [48]. Radiography experiments revealed that morphological changes of the lung begin as rapidly as two days post-infection. At necropsy, the edema of the lung was evidenced by a two times increase in lung weight. Viral isolates can be recovered from lung and tracheal tissues. Syncytial cells were observed in bronchial epithelium and endothelium of blood vessels although no viremia was detected. Many hemorrhages were also noted in the lung parenchyma, especially with HeV [48]. Lower doses also result in respiratory signs but are followed by neurological disabilities. In these animals, the lesions are widespread and can be found in heart, liver, kidney, bladder, brain, lung, and nasal epithelium [48]. RT-qPCR analysis revealed the presence of the virus in these organs, and also in urine. The lesions were largely represented by vasculitis but also inflammation in lung and brain where infected neurons were found [47]. 

Recent immunohistological evidence suggests that NiV is able to rapidly invade the central nervous system via olfactory route following an intranasal challenge [89]. The breach of the blood brain barrier, evidenced by an Evans Blue experiment, also reveals that the endothelium of the brain is permeable enough to let the virus circulate from systemic circulation to the brain [48]. Isolation of NiV is restricted to brain and kidney when animals are infected intranasally but is also possible from urine, spinal cord, lung, spleen, and liver when infected intraperitoneally [47]. Despite excretion of the virus in urine, transmission studies reveal that contamination by direct contact is not frequent and even less via fomites or aerosol routes [49]. 

Similarly to tests performed in ferrets [35], comparison of the pathogenicity of NiV isolates from Malaysia and Bangladesh was performed in hamsters. Results suggest that the Malaysia isolate has a faster replication rate and is more pathogenic than the Bangladesh isolate in which death is delayed. However, the clinical signs of the two isolates remain identical as well as viral distribution. In accordance with the accelerated pathology observed with the Malaysia isolate, the host immune response genes (especially IL-4, CXCL10, IL-6, TNFα and IFNγ) are activated earlier than for the Bangladesh isolate [50].

Overall, hamsters provide a significant improvement in understanding of henipaviruses immunopathogenesis. Their use has helped to determine the role of the non-structural proteins during the course of infection. Indeed, infection of hamsters with a recombinant virus lacking W protein remains highly lethal whereas recombinant viruses deficient for V or C proteins are nonpathogenic [51], reflecting the potential role of the V and C proteins in the dysregulation of the innate immune response. Furthermore, C-deficient NiV induces higher recruitment of inflammatory cells and less intensive histopathological lesions than wild-type NiV, in different organs of infected hamsters, thus suggesting the important role of NiV C protein in the regulation of early proinflammatory responses [52]. Finally, in agreement to the results obtained from transcriptomic analysis of NiV infected endothelial cells and patients succumbed to NiV-induced encephalitis, NiV infection in hamsters induces the important expression of proinflammatory chemokine CXCL10 in different organs [48,53,90]. This cytokine was confirmed to play an important role in HeV pathogenesis as well, and it was demonstrated that the development of neurological signs in both NiV and HeV infection coincided with tumor necrosis factor alpha and interleukin 1 production in hamsters [48,53].

Hamsters are also able to mount a good henipaviruses-specific seroneutralizing response after infection. They are thus extensively used to study potential vaccines [54,58], to monitor passive immunization assays [54,55,59] or to test new treatments [56,57] and they remain commonly used by different groups.

### 5.2. Guinea Pig

The Guinea Pig (*Cavia porcellus*) was the first susceptible rodent model developed to study infection with henipaviruses. However, due to high variability in results and development of new models, it lost its importance as a relevant animal model for the study of *Henipavirus* infection. Intradermal [60] and intranasal [25,60] inoculation were proven to be inefficient but subcutaneous injection of a high dose of HeV [34,60,61], or intraperitoneal injection of NiV [17], resulted in slight clinical signs usually associated with a systemic spreading of the virus revealed by immunohistochemistry, or did not show any clinical signs at all [47]. The observed clinical signs include depression, ruffled fur, and abnormal behavior followed by twitching, ataxia, and sudden death [17,60]. Necropsy revealed general cyanosis, congestion, and edema, especially in the lungs [34]. Histopathology and immunohistochemistry confirmed the presence of several syncytia and infected cells in the endothelium of blood vessels [34], lymphoid organs [91], brain, kidney, and transitional epithelium of the bladder [60,91], and myometrium and endometrium of the uterus [17,91]. Consistent with histological results, viral particles were isolated from heart, lung, kidney [17], urine, brain [60], uterus or placenta, and even from fetus [61]. If RT-qPCR allowed the detection of viral RNA in oral swabs [25], isolation of the virus from nasal, oral, rectal, and conjunctival swabs remained impossible [60]. Seroneutralizing antibodies are usually found in animals that survived [25,60]. Guinea Pigs were also used to analyze pathogenicity of the Cedar virus. Although they produced seroneutralizing antibodies 21 days after infection, they did not develop any visible clinical signs [4].

### 5.3. Mice

As murine models are very convenient to study viral infections and provide a well-developed experimental toolbox for analysis in immunovirology, the assessment of mice as a possible animal model for henipaviruses was undertaken rapidly after the discovery of both viruses. Despite possessing functional Ephrin (EFN) B2 and B3 virus receptors, mice were shown to be resistant to henipaviruses inoculation by intranasal [47], intraperitoneal [32,47], or subcutaneous [36] routes unless administered intracranially into suckling Balb/c mice [29]. Recently, two approaches were explored to create susceptible murine models of HeV and NiV infection.

#### 5.3.1. Aged Mice

Mice of different strains and ages were challenged with 50000 TCID_50_ of HeV either by intranasal or subcutaneous routes [46]. Results show that elderly mice (12 months old) from both strains (C57BL/6 or BALB/c) are susceptible to the disease when inoculated intranasally whereas subcutaneously injected mice and juveniles (eight weeks old) are resistant. Neurological symptoms resemble those observed in humans. They include ataxia, muscle tremors, hypersensitivity, and were followed by death. HeV antigen was only found in the brain, either by immunohistochemistry or by RT-qPCR, and seems to be restricted to neurons of the primary olfactory complex. Indeed, as demonstrated in hamsters [89] or in pigs [23], the central nervous system (CNS) can be reached without crossing the blood-brain-barrier via olfactory nerves after an intranasal inoculation of the virus. Histology analysis showed severe encephalitis characterized by neuronal degeneration, gliosis, perivascular cuffing, and associated to non-suppurative meningitis. Virus isolation was not possible from infected brains. In accordance with the RT-qPCR results, systemic lesions such as typical generalized vasculitis were totally absent in animals. However, a time-course study performed on aged BALB/c mice inoculated intranasally revealed that virus isolation was possible from lungs between day four and day 10, post infection, suggesting a transient infection of the lower respiratory tract. Seroneutralizing antibodies were sometimes detected, but at very low levels. The susceptibility of aged mice probably results from immunological changes that occur with aging. Indeed, defects of the adaptive immunity [92,93], as well as innate immune system, including macrophages functions [94], dendritic cells migration and interferon-α production by plasmacytoid dendritic cells [94] were all shown in old mice. 

#### 5.3.2. Transgenic Mice

Transgenesis in mice is a powerful and well-established approach that allows the addition of exogenous genes (transgenic and knock-in) or the inactivation of endogenous genes (knock-out, KO). Transgenic mice are important animal models as they make possible the functional assessment of different genes and have already provided remarkable insights in diverse aspects of viral pathogenesis. As the type I interferon (IFN-I) system is one of the first immune pathways triggered by viruses, mice lacking the IFN-I receptor (IFNAR KO) were generated in 1994 and shown to be sensitive to infection with VSV and LCMV, in contrast to wild type mice [95]. This murine model was used to evaluate the susceptibility to HeV and NiV infection as well [45].

IFNAR KO mice, backcrossed on C57BL/6 genetic background, were challenged with doses of NiV or HeV ranging from 100 pfu to 10^6^ pfu [45]. The three inoculation routes tested (intracranially, intraperitoneally, and intranasally) resulted in high mortality in IFNAR KO mice whereas only the intracranial route was fatal in WT mice; thus suggesting the important role of IFN-I signaling in the murine resistance towards henipaviruses. IFNAR KO mice showed clinical signs similar to those observed in humans and other animal models starting with agitation, lack of grooming, then followed by lordosis, expression of pain and discomfort, prostration, and finally locomotor disabilities and paralysis. All animals died between days six and nine when injected intraperitonally with 10^6^ pfu. 

**Figure 2:**
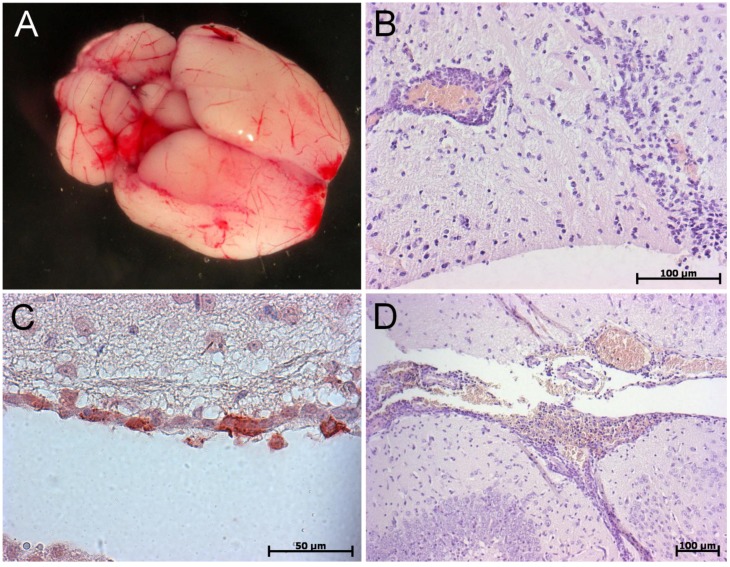
Gross pathology and immunohistology of IFNAR KO mice infected with NiV (**a**) Brain showing hemorrhages and congestion; (**b**) Vasculitis associated with leukocytes infiltration of the brain parenchyma; (**c**) Positive immunostaining of NiV antigens in the ependymal layer of a ventricle; (**d**) Hemorrhages, vasculitis and pronounced meningitis.

Gross pathology reveals congestion with scattered small hemorrhagic lesions in brain but also in liver and kidney, especially for NiV-infected animals (Figure 2). Histopathology shows that the brain is particularly affected with the presence of numerous spots of leukocyte infiltration, perivascular cuffing, hemorrhages, and non-suppurative meningitis (Figure 2A). Immunohistochemistry (IHC) analysis confirmed the presence of the virus in neurons and ependymal cells. In the lungs, intense inflammation with edema, focal necrotizing alveolitis and vasculitis was noticed and antigen presence was confirmed by IHC (Figure 2B-D). As a particularity of this model, hepatic lesions are severe with NiV infection and result in focal necrosis, vasculitis, and presence of several syncytial cells. The amount of viral RNA was quantified by RT-qPCR and revealed that all sampled organs (brain, lung, spleen, and liver) were positive with the highest quantity in lungs. Hepatic lesions are also observed to a lesser extent in hamsters [47] and NiV RNA was found in liver of ferrets [38] and African green monkeys [41]. Such observations suggest that IFN is a potent controller of pathogenesis in the liver.

IFNAR KO mice sub-lethally infected either by NiV or HeV develop a high titer of seroneutralizing antibody response within three weeks, demonstrating thus their capacity to efficiently mount virus-specific humoral immune response. This new model suggests the critical role of type I signaling in the control of HeV and NiV infection and provides the possibility to use numerous and powerful tools available for mice to further study henipaviruses pathogenesis, prophylaxis, and treatment.

## 6. Conclusions and Perspectives

The changing ecological pressures on flying foxes owing to deforestation, urban development, altered foraging, and behavioral patterns all play a role in the continuing reemergence of *Henipavirus* infections. Since 1994, animal models have provided significant advances in fundamental research into henipaviruses and given critical support to discover and assess potential prophylactic and therapeutic treatments. Future challenges in this field will include the comprehension of the mechanisms allowing the efficient control of viral replication in fruit bats, which can give rise to original therapeutic approaches. The recent discovery of the third nonpathogenic member of *Henipavirus* genus, the Cedar virus, offers new possibilities for comparative analysis of henipaviruses immunopathogenesis. The use of appropriate laboratory animal models is critical for correct studies and further development of antiviral therapeutics and vaccines. As showed in Table 1, the choice of the most accurate animal model strongly depends of the goal of the study, and should be done in accordance with the particularities and limitations of each model. The recent discovery of susceptibility of genetically modified mice to *Henipavirus* infection opens new avenues in the field of the immunobiology of these viruses. The use of a small animal model for which many experimental tools are available should improve our understanding of the control of this highly lethal infection and will provide significant help to develop new treatments and prevention.
